# EV71 Infection Induces IFNβ Expression in Neural Cells

**DOI:** 10.3390/v11121121

**Published:** 2019-12-04

**Authors:** Hsing-I Huang, Jhao-Yin Lin, Sheng-Hung Chen

**Affiliations:** 1 Research Center for Emerging Viral Infections, College of Medicine, Chang Gung University, Kwei-Shan, Tao-Yuan 33303, Taiwan; jhaoyinlin@gmail.com (J.-Y.L.); simon865712000@gmail.com (S.-H.C.); 2 Department of Medical Biotechnology and Laboratory Science, College of Medicine, Chang Gung University, Kwei-Shan, Tao-Yuan 33303, Taiwan; 3 Graduate Institute of Biomedical Sciences, College of Medicine, Chang Gung University, Kwei-Shan, Tao-Yuan 33303, Taiwan; 4 Department of Pediatrics, Chang Gung Memorial Hospital, Linkou 33303, Taiwan

**Keywords:** EV71, IFNβ, neural cells, pattern recognition receptor, interferon-stimulated genes

## Abstract

Enterovirus 71 (EV71) can invade the central nervous system (CNS) and cause neurological disease. Accumulating evidence indicates that EV71 can directly infect neurons in the CNS. Innate immune responses in the CNS have been known to play an essential role in limiting pathogen infections. Thus, investigating the effects of EV71 infection of neural cells is important for understanding disease pathogenesis. In this study, human neural cells were infected with EV71, and interferonβ (IFNβ) expression was examined. Our results show that IFNβ expression was upregulated in EV71-infected neural cells via pattern recognition receptors (PRRs) sensing of virus RNA. The PRRs Toll-like receptor 3 (TLR3), Toll-like receptor 8 (TLR8), and melanoma differentiation-associated gene-5 (MDA-5), but not retinoic acid-inducible gene-I (RIG-I) and Toll-like receptor 7 (TLR7), were found to be EV71-mediated IFNβ induction. Although viral proteins exhibited the ability to cleave mitochondrial antiviral signaling protein (MAVS) and Toll/IL-1 receptor (TIR) domain-containing adaptor-inducing IFN-β (TRIF) in neural cells, levels of viral protein expression were low in these cells. Furthermore, neural cells efficiently produced IFNβ transcripts upon EV71 vRNA stimulation. Treating infected cells with anti-IFNβ antibodies resulted in increased virus replication, indicating that IFNβ release may play a role in limiting viral growth. These results indicate that EV71 infection can induce IFNβ expression in neural cells through PRR pathways.

## 1. Introduction

Enterovirus 71 (EV71) is a member of the family *Picornaviridae*, which is composed of non-enveloped single-stranded RNA viruses. EV71 has been recognized for its ability to invade the central nervous system (CNS) and cause neurological symptoms. Data from clinical and animal studies suggest that after infection EV71 viral capsid proteins are present in neural cells in the brain, thus providing evidence that EV71 can directly infect neurons [1,2]. In the brains of EV71-infected monkeys, EV71 antigen could be detected in the thalamus and motor cortex [1]. Furthermore, EV71 RNA and protein expression could be detected in the brain neurons from deceased EV71-infected patients [2]. However, the effects of EV71 infection in host neural cells remain to be identified.

Antiviral innate immune responses have been shown to play essential roles in defending cells against viral infections. Type I interferons (IFNs), the key regulators of innate immunity, are produced by cells in response to viral sensing. Once secreted, IFNs interact with their receptors resulting in the expression of interferon-stimulated genes (ISGs), which function to suppress viral replication and regulate the inflammatory process [3,4]. For example, a previous study showed that IFNβ may have important functions in controlling the replication of enteroviruses [5]. IFNβ has also been demonstrated to be effective in decreasing the virus yield in coxsackeivirus B3 (CVB3)-infected myocardial fibroblasts [6]. In another study, mice treated with IFN inducers before EV71 infection showed increased survival rates with decreased viral loads in brain/muscle tissue [7]. Nonetheless, conflicting results have been obtained when interferon was administered after EV71 infection, with mortality increasing in one study [8]. Therefore, it has been suggested that interferon plays different roles during different phases of EV71 infection.

Viral pathogens can be detected through pattern recognition receptors (PRRs), including Toll-like receptors (TLRs), NOD-like receptors (NLRs), retinoid acid-inducible gene I (RIG-I), and melanoma differentiation-associated gene 5 (MDA-5). These receptors induce IFNβ expression by activating TIR domain-containing adaptor-inducing IFN-β (TRIF) and mitochondrial antiviral signaling protein (MAVS), which form a complex with TANK-binding kinase (TBK), IκB kinase ε (IKKε), and interferon regulatory factors (IRFs) [9]. Subsequent activation of IRFs results in expression of type I IFNs and proinflammatory cytokines [10]. However, not all infected cells can produce IFNs because certain viruses can subvert cellular IFN induction pathways [11]. For example, some viral proteins, including influenza virus proteins PB1-F2 and PB2-S1, interact with the mitochondrial protein MAVS to inhibit the induction of IFN production [12,13]; other viruses such as hepatitis C virus and dengue virus suppress IFN activation by cleaving MAVS and Stimulator of interferon genes (STING), respectively [14,15,16].

Accumulating evidence indicates that the initiation of innate immune responses is regulated in a cell type-specific manner. Different cell types are equipped with specific PRR patterns to sense invading pathogens. Previous studies have demonstrated that some viruses such as dengue virus can induce IFNβ and ISG production in the brain; in contrast, IFNs are not detectable in infected dendritic cells, which suggests that IFN induction is disparately regulated in different cells [17,18]. In the brain, TLR2 is expressed by astrocytes, whereas TLR1 and TLR9 expression is limited to infiltrating immune cells [19]. RIG-I-like receptors (RLRs), including RIG-I, MDA-5, and laboratory of genomics and physiology 2 (LGP2), are widely expressed in most tissues. IFNs can also be generated by neurons [20]; neurons express TLR3, and poly(I:C) alone can induce expression of IFNβ in NT2-differentiated neurons [21]. In addition, RNA viruses such as rabies virus have been shown to evoke IFNβ expression in neuronal cells [21]. Furthermore, a recent study showed that the brains of Theiler’s murine encephalomyelitis virus (TMEV) infected mice containing type I IFN mRNA during the acute phase of encephalitis [22], indicating that enteroviruses might be able to activate IFN production.

Recent studies have demonstrated that EV71 can actively replicate in neural lineage ranging from neuroblastoma cells to primary neurons [23,24]. Regardless, it is not completely clear whether EV71 evokes innate immune responses in neural cells. Although EV71 has been demonstrated to inhibit IFNβ induction by affecting the pathways mediated by RIG-I, TLR3, and MDA-5 via viral proteases [25,26], animal experiments have indicated that IFNβ expression is enhanced in the brain tissue of EV71-infected mice [27]. Therefore, we hypothesized that EV71 infection may trigger activation of IFN expression in the CNS. Our results also demonstrate that EV71 can induce IFNβ expression in neural cells and that this increased IFNβ expression is associated with TLR3, TLR8, and MDA5. Furthermore, ISGs are upregulated in EV71-infected neural cells, indicating that these cells can respond to secreted IFN.

## 2. Materials and Methods

### 2.1. Cells and Viruses

EV71 strain TW-2231 (EV71 2231) (subgenotype C2) and EV71 strain BrCr (subgenotype A) were used in this study. Unless otherwise stated, cells were infected with EV71 TW-2231. RD (human rhabdomyosarcoma) cells were grown in Dulbecco’s modified Eagle medium (DMEM) containing 10% fetal bovine serum (FBS), 1% non-essential amino acid, 1% L-Glutamine, and 1% penicillin/streptomycin (all from Thermo-Fisher Scientific, Waltham, MA, USA). SF268, SH-SY5Y, and IMR32 cells were obtained from Bioresource Collection and Research Center, Taiwan. SF268 cells are defined as human malignant glioblastoma cells, while IMR32 and SH-SY5Y cells were derived from human neuroblastoma. These cells were maintained in DMEM supplemented with 10% fetal bovine serum (FBS) and 1% L-Glutamine (all from Thermo-Fisher Scientific, Waltham, MA, USA). Human neural stem cells (hNSCs) were obtained from commercial sources (HUXNF-01001, Cyagen Biosciences or U7800-100, Thermo-Fisher Scientific, Waltham, MA, USA). The human neural stem cells were cultivated in a neural stem cell medium containing neural stem cell supplements. For neuronal differentiation, hNSCs were plated in the CELLStart coated plate and incubated for 2 days. The culture medium was then changed to knockout DMEM/F12 with 2% StemPro Neural Supplement and 1% Glutamax, and incubated for 5–7 days at 37 °C and 5% CO_2_. The differentiation was confirmed by detecting the expression of neuron-specific markers including MAP-2 and Neuron-specific class III β-tubulin. All cells were maintained in a 37 °C humidified incubator equilibrated with 5% CO_2_.

### 2.2. Viral Infection

Cells were seeded on 12-well plates at the concentration of 2 × 10^5^ cells per well. The cells were washed by PBS once after overnight seeding. Virus was then added at specified multiplicity of infection (MOI) with serum-free DMEM. After one hour of adsorption, the virus-containing medium was decanted and DMEM containing 2% FBS was then added. To inactivate EV71, virus stock was kept on ice and exposed to UV light in UV crosslinker (Spectronics corporation, NY, USA) at 2 × 10^5^ μJ/cm^2^ for 20 min.

### 2.3. Reagents

Poly(I:C) (Sigma-Aldrich, St. Louis, MO, USA) was prepared using PBS. Motolimod (Selleckchem, Houston, TX, USA) were prepared at 1 mM in DMSO. Poly(I:C)_HMW/LyoVec^TM^ (InvivoGen, San Diego, CA, USA) was prepared at 0.125 μg/mL in endotoxin-free water. Poly(A:U) (InvivoGen, San Diego, CA, USA) was prepared at 1 μg/mL in sterile physiologic water. The cells were seeded in 12-well plates and incubated overnight. Lipofectamine 2000 (Thermo-Fisher Scientific, Waltham, MA, USA) was used for transfection and reagents A and B were prepared according to the manufacturer’s protocol. Reagent A contained 100 μL opti-MEM with EV71 RNA, 1 μg poly(I:C), 1 μg poly(I:C)_HMW, 1 μg poly(A:U), or 10 μM motolimod. Reagent B contained 100 μL opti-MEM with 2 μL Lipofectamine 2000. Reagents A and B were mixed and incubated at room temperature for 20 min. The mixtures were then added into tested cells for transfection.

### 2.4. Immunofluorescence Staining

Cells were fixed with ice-cold 4% paraformaldehyde for 15 min at room temperature. After being washed by 1× PBS three times, the fixed cells were then permeabilized by addition of 0.5% triton X-100 for another 5 min. After being washed by PBS three times, the cells were then blocked by PBS containing 2% FBS for 1 hour at room temperature. After blocking, the cells were incubated with primary antibodies: mouse anti-EV71 3D (1:500, Genetex, Irvine, CA, USA), rabbit anti-MAP2 (1:200, Millipore, Burlington, MA, USA), TUJ1 (mouse anti-neuron-specific class III β-tubulin)(1:200, Millipore, Burlington, MA, USA), and rabbit anti-phosphorylated IRF3-Ser396 (1:200, Cell Signaling Technology, Danvers, MA, USA) at 4 °C overnight. The cells were then washed three times with 1× PBS and incubated with Dylight 594 conjugated donkey anti-mouse secondary antibody or Dylight 488 conjugated goat anti-rabbit secondary antibody (1:1000, Jackson ImmunoResearch Laboratories, West Grove, PA, USA) for 1 h at room temperature. The cells were washed three times with 1× PBS, and cell nuclei were counterstained with DAPI (4′,6-diamidino-2-phenylindole) (Sigma-Aldrich, St. Louis, MO, USA). The images were collected by a fluorescence microscope (Olympus BX51, Olympus, Tokyo, Japan).

### 2.5. Protein Isolation and Western Blot

Total protein was extracted from mock- and EV71-infected cells. Protein samples were separated by 8% or 12% SDS-polyacrylamide gel electrophoresis and then transferred onto a polyvinylidene fluoride membrane (PVDF) (GE Healthcare Life Sciences, Boston, MA, USA). The protein-containing membranes were blocked with 5% skim milk in Tris-buffered saline Tween-20 (TBST, 20 mmol/mL Tris-HCl, pH 7.4, 150 mmol/L NaCl, and 0.1% Tween-20) at room temperature for 1 h. The membrane was then incubated with primary antibodies: mouse anti-EV71 3D (1:2000, Genetex, Irvine, CA, USA), mouse anti-EV71 3C (1:500, a generous gift from Dr. Shin-Ru Shih, Chang Gung University), mouse anti-EV71 VP (1:2000, Millipore, Burlington, MA, USA), anti-IRF3 (1:1000, Santa Cruz Biotechnology, Dallas, TX, USA), anti-phosphorylated IRF3-Ser396 (1:1000, cell signaling, Danvers, MA, USA), rabbit anti-TRIF (1:1000, cell signaling, Danvers, MA, USA), mouse anti-MAVS (1:1000, Santa Cruz, Dallas, TX, USA), mouse anti-TLR3 (1:1000, Abcam, CAMB, UK), mouse anti-TLR8 (1:1000), rabbit anti-TLR7 (1:1000, both from Thermo-Fisher Scientific, Waltham, MA, USA), rabbit anti-MDA5 Ab (1:2000, Enzo Life Sciences, Farmingdale, NY, USA), rabbit anti-RIG-I (1:1000, Pro-Sci, San Diego, CA, USA), and mouse anti-β-actin (1:20,000, Sigma-Aldrich, St. Louis, MO, USA). Subsequently, the membrane was probed with anti-mouse or anti-rabbit secondary antibody conjugated with horseradish peroxidase (1:5000, Jackson ImmunoResearch Laboratories, St. Louis, USA). The protein was detected with a chemiluminescence reagent (PerkinElmer, Waltham, MA, USA) and a Chemi^TM^ imaging system (Bio-rad, Hercules, CA, USA).

### 2.6. RNA Isolation and RT-PCR

The total RNA was collected by TRI reagent^TM^ solution (Thermo-Fisher Scientific, Waltham, MA, USA) at various times. The cells were homogenized by TRI reagent solution and mixed with chloroform. The homogenate was incubated for 5 min at room temperature and centrifuged at 12,000× *g* for 15 min at 4 °C. The aqueous phase was transferred to fresh tubes. The aqueous phase containing RNA was added with an equal amount of isopropanol and incubated at room temperature for 10 minutes. The mixture was centrifuged at 12,000× *g* for 10 min at 4 °C and the supernatant was removed. The RNA pellet was washed by 1 ml 75% ethanol at 7000× *g* for 5 min at 4 °C. The 75% ethanol was removed and the RNA pellet was air dried at room temperature. The RNA pellet was then dissolved by sterile water. One microgram of total RNA was used for cDNA synthesis. The synthesis of cDNA was performed with using RevertAid First Strand cDNA Synthesis Kit (Thermo-Fisher Scientific, Waltham, MA, USA). One μL of cDNA sample with 5 μM primers was performed for the qPCR and SYBR green (KAPA Biosystems, Wilmington, MA, USA) was used as the quantifying expression. qPCR assay was carried out in a 384-well plate and analyzed by Roche Lightcycle 480 (Roche, Basel, SW). Each sample was assayed in triplicates and 18S rRNA was used as a reference gene. The relative quantification of each gene was analyzed by 2^−∆∆CT^ method. The primers were designed according to the gene sequence published in NCBI (Table 1).

### 2.7. siRNA Knockdown

The cells were seeded in 12-well plates and incubated overnight. The siRNAs specific for TLR3, TLR7, TLR8, RIG-I, and MDA-5, as well as scrabble siRNA, were used in this study (all from Sigma-Aldrich, St. Louis, MO, USA). The stock of siRNA was prepared in 100 μM with RNase-free distilled water and the working concentration was 100 nM. Lipofectamine 2000 RNAiMAX (Thermo-Fisher Scientific, Waltham, MA, USA) was used for siRNA transfection, and reagent A and B were prepared. Reagent A contained siRNA diluted in 100 μL opti-MEM and reagent B contained 4 μL lipofectamine 2000 RNAiMAX diluted in 100 μL opti-MEM. Reagent A was added to reagent B and incubated for 5 min at room temperature. The cells were washed once with PBS and fresh culture medium was added to the cells. The mixture of reagent A and B was added to the cells and incubated at 37 °C with 5% CO_2_ for 72 h. 

### 2.8. In Vitro Proteinase Cleavage Assay

The protein extracts of SF268 and 293T cells were prepared upon treatment with CA630 lysis buffer (1% CA630, 50 mM Tris-base, 150 mM NaCl, pH8.0, without protease inhibitor) for 30 min on ice. The cells were harvested and centrifuged at 13,000 rpm for 10 min at 4 °C and the supernatants were collected. 30 μg of SF268 or 293T protein extract was incubated with 15 μg of viral proteinase EV71 3C or EV71 3C^C147S^ (kindly provided by Dr. Shin-Ru Shih, Chang Gung University, Taiwan) and cleavage buffer (50 mM Tris-HCl, 50 mM NaCl, 5 mM DTT and 1 mM EDTA, pH 7.5) at a total volume of 15 μL. The mixture was incubated for 4 h at 37 °C and the signal of proteolytic cleavage was analyzed by immunoblotting.

### 2.9. IFN-β Antibody Blocking Assay

SF268 was seeded on 12-well plates at the concentration of 2.5 × 10^5^ cells/well. After incubation overnight, the cells were infected with EV71 at an MOI of 40 and then IFN-β antibody (Thermo-Fisher Scientific, Waltham, MA, USA) was added in the fresh DMEM containing 2% FBS after virus adsorption. The total RNA was harvested at 24 h post infection and RT-qPCR was performed to detect viral replication.

### 2.10. Plaque Assay

The viruses were harvested at different time points and then quantified by plaque assay. RD cells were expanded in DMEM/10% FBS and seeded on 6-well plates at the concentration of 5 × 10^5^ cells/well. After incubation overnight, the cells were infected by serially diluted virus solution. After one hour of adsorption, the virus suspension was decanted and replaced by DMEM (supplemented with 2% FBS and 0.3% agarose). After 96 h, the medium was removed and the cells were stained by crystal violet solution.

### 2.11. Statistical Analysis

Results were expressed as the mean ± standard deviation. Statistical significance was determined by Student’s two-tailed t-test. Statistical significances are indicated as follows: *, *p* < 0.05, **, *p* < 0.01, ***, *p* < 0.001.

## 3. Results

### 3.1. EV71 Induces IFNβ Expression in Neural Cells

**Figure 1:**
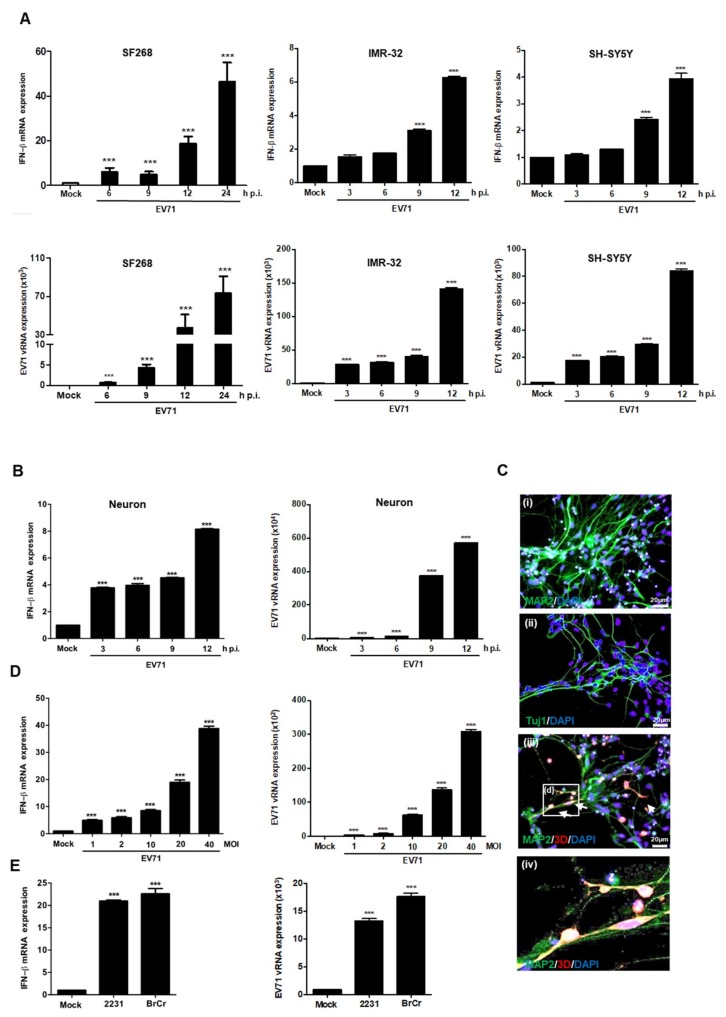
Enterovirus 71 (EV71) induces the expression of IFNβ in neural cells. (**A**) SF268, IMR32, and SH-SY5Y cells were infected with EV71 at an multiplicity of infection (MOI) of 40, and the expression levels of IFNβ and EV71 vRNA were examined by RT-qPCR at different time points. (**B**) Human neural stem cells (hNSC)-derived neurons were infected with EV71 at an MOI of 40, and the expression levels of IFNβ and EV71 vRNA were examined by RT-qPCR at different time points. (**C**) Human NSCs were differentiated into neurons and the expression of MAP2 (i) and Neuron-specific class III beta-tubulin (ii) was assessed by immunofluorescence staining (magnification = 200x). The differentiated neurons were then infected with EV71 at an MOI of 40 for 24 h and double immunofluorescence staining was applied to detect EV71 infection. Arrows point to the cells expressing EV71 3D antigen (red) and MAP2 (green)(iii); a higher magnification of white box is shown (iv). (**D**) SF268 cells were infected with EV71 at different MOIs for 12 h, total RNA was extracted, and RT-qPCR was applied to detect the expression levels of IFNβ and vRNA. (**E**) SF268 cells were infected with EV71 2231 and EV71 BrCr at an MOI of 40 for 12 h, and the expression of IFNβ and vRNA was detected by RT-qPCR. Asterisks indicate values that are with statistically significant compared to mock-infected cells (*, *p* < 0.05, **, *p* < 0.01, ***, *p* < 0.001).

To examine whether EV71 infection was sufficient to induce IFNβ expression in neural cells, human glioblastoma cell line (SF268) and neuroblastoma cell lines (IMR32 and SH-SY5Y) were cultured and infected with EV71 at a multiplicity of infection (MOI) of 40, and the infected cells were harvested at different time points. RT-qPCR analysis revealed that the expression levels of IFNβ increased in a time-dependent manner (Figure 1A). To examine whether IFNβ expression is upregulated in differentiated neuronal cells, we examined the expression of IFNβ in mock- and EV71-infected human NSC-derived neuronal cells. RT-qPCR analysis revealed that IFNβ transcripts were also upregulated in EV71-infected differentiated neurons (Figure 1B). Immunofluorescence staining was applied to examine the expression of neuron-specific markers MAP2 and neuron-specific class III β-tubulin to confirm differentiation (Figure 1C). EV71 infection was confirmed by detecting the presence of virus 3D in MAP2 positive neurons (Figure 1C). SF268 cells were chosen for subsequent experiments because EV71 infection is able to induce more IFNβ transcripts in these cells. Expression of the EV71 5’ untranslated region (UTR) was used to confirm EV71 infection and upregulation of IFNβ expression occurred in a dose-dependent manner (Figure 1D). Different EV71 strains, including 2231 and BrCr, were used to infect SF268 cells at an MOI of 40 for 12 h, and according to RT-qPCR, all tested viruses were able to induce expression of IFNβ (Figure 1E). Taken together, our results show that IFNβ expression is increased in various neural cell types upon EV71 infection.

### 3.2. EV71 Induces IRF3 Phosphorylation in Neuronal SF268 Cells

**Figure 2:**
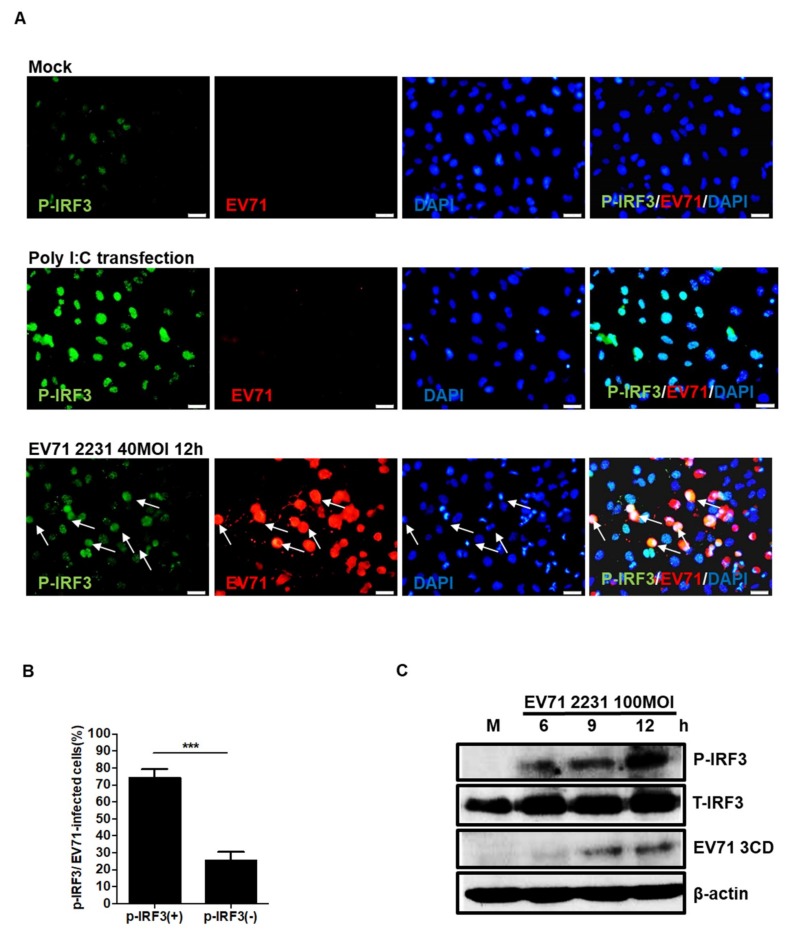
EV71 infection induces IRF3 phosphorylation. Cells were seeded and infected with EV71 at an MOI of 40 for 12 h. (**A**) Expression of pIRF3 and viral protein 3D was examined by double immunofluorescence staining. Mock-infected cells were used as negative controls; cells transfected with poly(I:C) were applied as positive controls (magnification = 200×). Arrows indicate the cells were positive stained with anti-EV71 3D and anti-pIRF3 antibodies. (**B**) Percentages of pIRF3-positive and negative cells were counted in EV71-infected cells. (**C**) Cells were infected with EV71 at an MOI of 100. Western blot analysis was performed to detect expression of total IRF3 (T-IRF3), phosphorylated IRF3 (P-IRF3), and viral protein 3CD. Expression of β actin was used as a control. Asterisks indicate values that are statistically significant compared to mock-infected cells (*, *p* < 0.05, **, *p* < 0.01, ***, *p* < 0.001).

Previous studies have shown that IRF3 phosphorylation plays an essential role in the viral induction of IFNβ in cultured cells [28]. Furthermore, IRF3 has been demonstrated to be important in protecting the brain from ischemic injury [29]. We next sought to examine whether IRF3 is phosphorylated in SF268 cells in response to EV71 infection. Double immunofluorescence staining of mock- and EV71-infected cells was used to reveal the localizations of the EV71 3D protein and phosphorylated IRF3 (pIRF3) in infected SF268 cells (Figure 2A), and pIRF3 was detected in the nucleus of the infected cells. The percentages of double and single positive cells were counted and the results were as shown (Figure 2B). We noticed that some EV71 infected cells were absent for pIRF3 expression. The time needed for virus antigen expression and IRF3 phosphorylation may differ and not all infected cells were at the same stage of infection. Therefore, the phosphorylation of IRF3 cannot be detected in all viral antigen positive cells. Additionally, total protein was extracted from the infected cells at different time points, and the levels of pIRF3 expression were determined by western blotting. Our results indicated that the amount of pIRF3 increased in a time-dependent manner (Figure 2C).

### 3.3. EV71 Cleaves TRIF and MAVS in SF268 Cells

**Figure 3:**
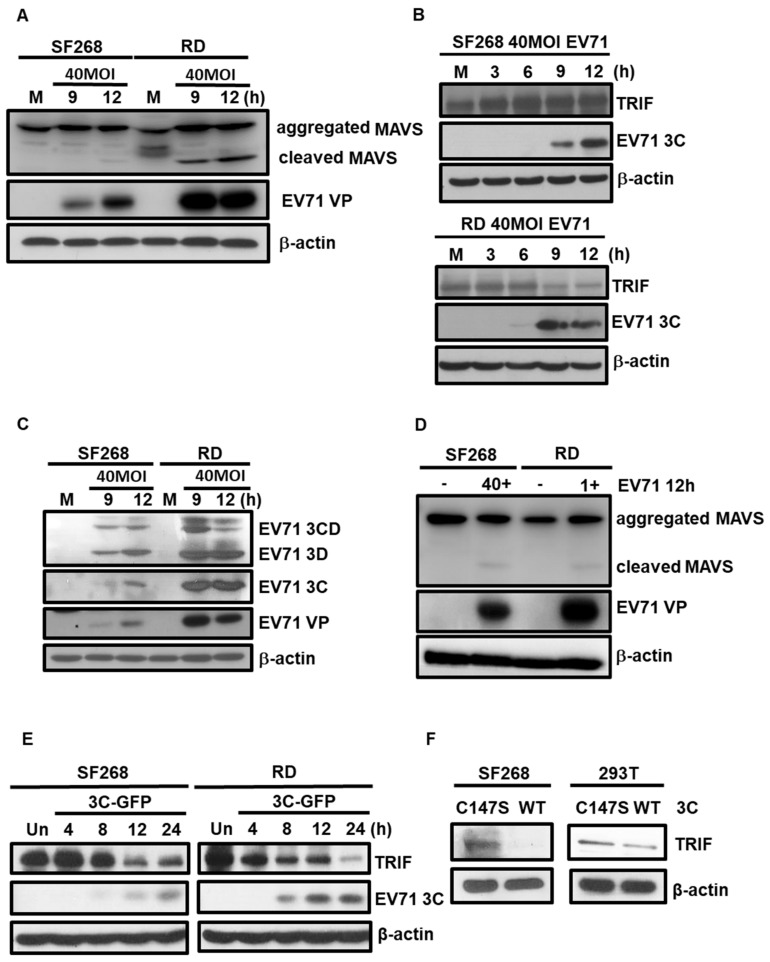
MAVS and TRIF proteins are cleaved in EV71 infected SF268 and human rhabdomyosarcoma (RD) cells. SF268 and RD cells were infected with EV71 at an MOI of 40. (**A**) Expression levels of MAVS, EV71 VP, and β actin were assessed by western blot. (**B**) Protein levels of TRIF, EV71 3C, and β actin were examined by immunoblot analysis. (**C**) Protein lysates were harvested at indicated times from SF268 and RD cells that were mock-infected or infected with EV71. Expression levels of virus protein 3CD, 3D, 3C, and VP were assessed by immunoblot analysis. (**D**) SF268 and RD cells were infected with EV71 at MOIs of 1 and 40, respectively. After 12 h of infection, expression of MAVS, EV71 VP, and β actin was analyzed by western blotting. (**E**) SF268 and RD cells were transfected with 3xflag-TRIF-myc and 3C-EGFP plasmids. Immunoblot analysis was performed to detect the expression levels of TRIF and 3C. (**F**) In vitro cleavage assays were performed to assess the activities of 3C ^WT^ and 3C^C147S^ in cleaving TRIF proteins in SF268 and 293T cells. The expression of β actin was used as an internal control.

SF268 and RD cells were infected with EV71 at the MOI of 40, and the expression levels of MAVS and EV71 VP were detected by western blotting. The results revealed that MAVS protein was cleaved in EV71 infected RD cells after nine hours of infection. However, the cleaved protein was hardly to be detected in EV71-infected SF268 cells (Figure 3A). Similar results were observed in TRIF protein: the expression levels were significantly degraded in RD cells, but not in SF268 cells (Figure 3B). Previous studies reported that EV71 can inhibit induction of the IFN response through the 2A and 3C proteases, which cleave MAVS and TRIF, respectively, and the activities of viral proteases have been used to explain why IFNβ is not upregulated in EV71-infected RD and HeLa cells [26,30]. As the absence of degradation of MAVS and TRIF in SF268 cells at early stages of infection, we postulated that the difference could be due to the low viral protein levels in these cells. To test this, SF268 and RD cells were infected with EV71 at the same MOI, and expression levels of various viral proteins were examined by immunoblotting analysis. Our results revealed that much more viral proteins were synthesized in RD cells compared to SF268 cells (Figure 3C). Nevertheless, it is possible that the protease activities of viral proteins could be affected by cellular environment. To evaluate whether the ability of viral proteins to cleave TRIF and MAVS proteins is cell type-dependent, SF268 and RD cells were infected with EV71 at MOIs of 40 and 1, respectively. Western blotting was performed to detect expression levels of MAVS and viral protein. Based on our results, EV71 is able to cleave MAVS in SF268 cells with an efficiency similar to that observed in RD cells (Figure 3D). Next, SF268 cells were transfected with 3xflag-TRIF-myc and then with 3C-EGFP, and TRIF and 3C expression was analyzed by immunoblotting. Levels of TRIF protein were decreased in SF268 cells at 12 hours after transfection of the 3C-containing plasmid (Figure 3E). However, in RD cells, TRIF expression was drastically decreased at eight hours post-transfection, which might be explained by the distinct transfection efficiencies of different cell types. An enzymatic digestion assay was also applied to assess whether SF268 cells contain proteins that block the action of EV71 3C. Purified wild type (WT) 3C^pro^ and mutant 3C^pro^ (C147S) were incubated with lysates harvested from SF268 and 293T cells, and western blotting results showed that TRIF expression was affected by WT 3C^pro^, but not the mutant 3C^pro^(C147S) (Figure 3F), suggesting that SF268 cell lysates did not contain components that inhibit the activity of the viral 3C protease. Our results revealed that viral proteases can cleave MAVS and TRIF in both cell types. Thus, EV71-induced IFNβ expression in SF268 cells is not involved with the cellular factors that affect protease activity in cleaving TRIF and MAVS.

### 3.4. EV71 RNA Induces IFNβ Expression via TLR3, TLR8, and MDA-5

**Figure 4:**
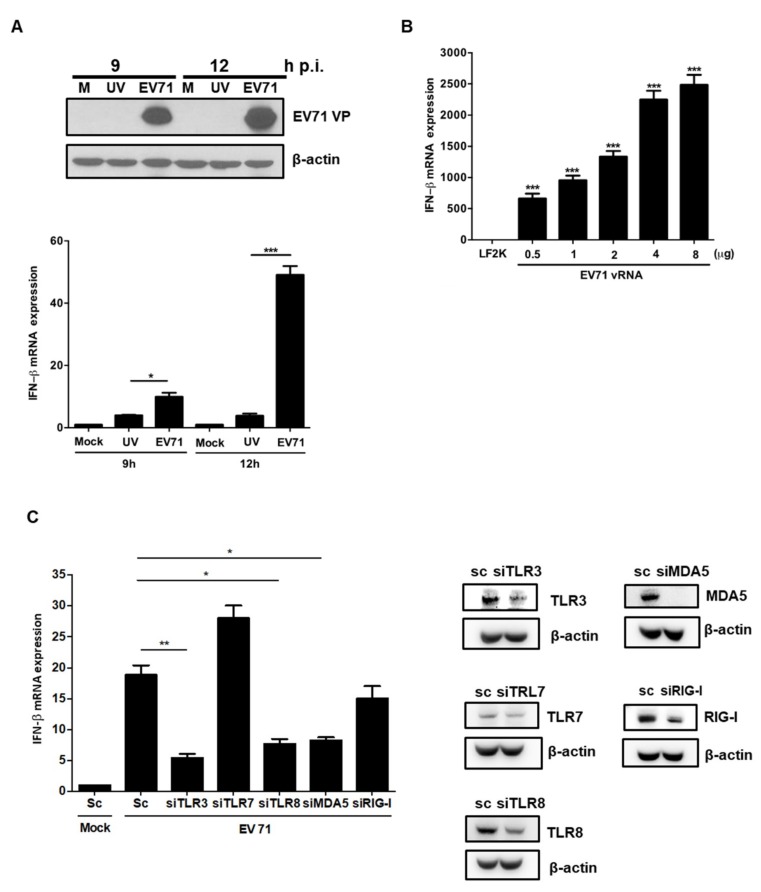
TLR3, TLR8, and MDA-5 are responsible for inducing IFN expression. (**A**) SF268 cells were infected with EV71 or UV-treated EV71 for 9 h and 12 h. Western blotting was performed to detect expression of EV71 VP and βactin, which was applied as an internal control. IFNβ transcript expression was examined by RT-qPCR. (**B**) Different amounts of EV71 viral RNA were transfected into SF268 cells for 12 h and expression of IFN was analyzed by RT-qPCR (LF2K= lipofectamin 2000). (**C**) SF268 cells were transfected with scrambled or siRNA against TLR3, TLR7, TLR8, MDA5, and RIG-I. After 48 h, the cells were infected with EV71 at an MOI of 40 for 12 h. RT-qPCR analysis was then applied to detect the expression levels of IFNβ. The inhibition efficiency of specific siRNAs was examined by western blotting (sc = scramble). Asterisks indicate values that are statistically significant compared to control cells (*, *p* < 0.05, **, *p* < 0.01, ***, *p* < 0.001).

IFNβ expression was not upregulated in SF268 cells treated with UV-irradiated EV71 viral particles, which cannot actively replicate in host cells (Figure 4A). This observation suggests that the binding of viral particles to receptors is not sufficient to induce type I IFN expression. To determine whether viral RNA (vRNA) can induce IFNβ, EV71 vRNA was transfected into SF268 cells, and IFNβ transcript levels were measured. RT-PCR results showed that the expression levels of IFNβ were increased in a dose-dependent manner (Figure 4B). To further identify which PRRs are responsible for the EV71-induced type I IFN response, we performed a knockdown experiment by transfecting siRNAs specific for TLR3, TLR7, TLR8, RIG-I, and MDA5 and then analyzed IFNβ expression by RT-PCR after EV71 infection. Knockdown efficiencies were confirmed by western blotting and our results demonstrated that TLR3, TLR8, and MDA-5 play essential roles in mediating upregulation of IFNβ expression upon EV71 infection (Figure 4C). 

### 3.5. EV71 vRNA Causes Higher IFNβ Expression in SF268 Cells Than in RD Cells

**Figure 5:**
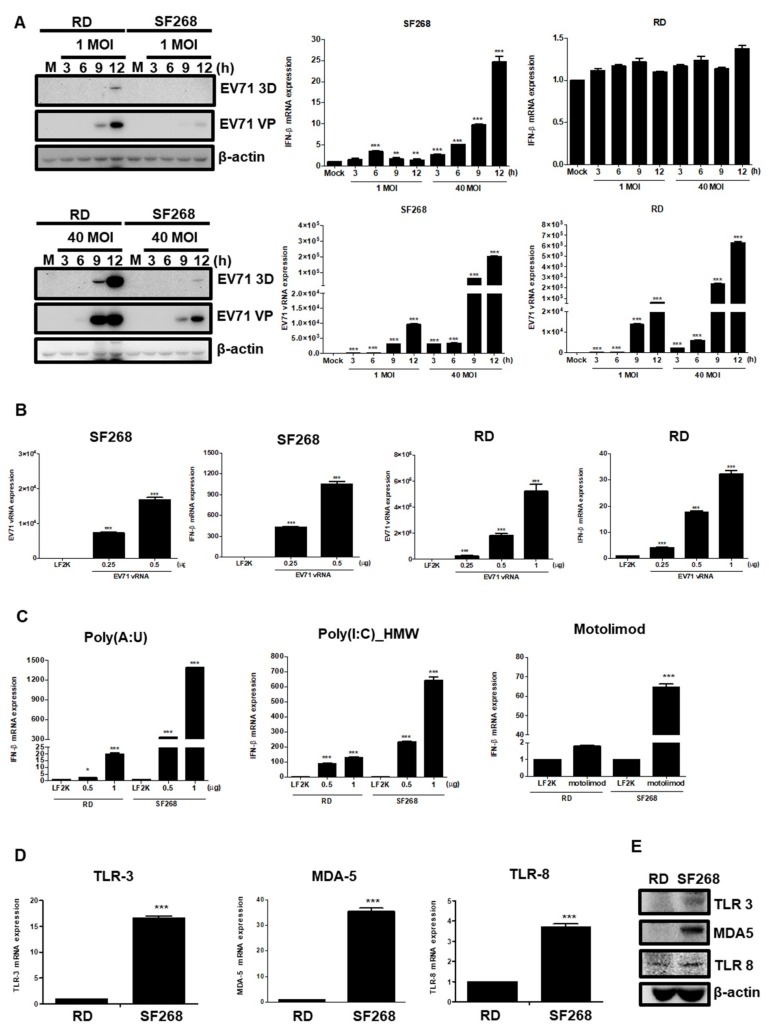
Transfection of EV71 viral RNA induces more IFN transcripts in SF268 cells than in RD cells. SF268 and RD cells were infected with EV71 at MOIs of 1 and 40, as indicated, and samples were collected at different time points. (**A**) Total protein was isolated and subjected to immunoblotting to detect expression of EV71 viral protein 3D and VP; β actin was used as an internal control. RT-qPCR was performed to detect the expression levels of EV71 vRNA and IFNβ. (**B**) EV71 genomic RNA (0.5 and 0.25 μg) was transfected into SF268 and RD cells, respectively, and the expression levels of EV71 vRNA and IFNβ were examined by RT-qPCR. (**C**) RD and SF268 cells were transfected with poly(A:U), poly(I:C)_HMW, and motolimod with lipofectamine 2000. Total RNA was collected at 12 h post transfection and RT-qPCR was performed to detect the expression levels of IFNβ. (LF2K = lipofectamine 2000) (**D**) RT-qPCR was performed to assess the expression levels of TLR3, TLR8, and MDA-5. (**E**) The expression levels of TLR3, MDA-5, and TLR8 were assessed by western blotting. Asterisks indicate values that are statistically significant compared to mock-infected cells (*, *p* < 0.05, **, *p* < 0.01, ***, *p* < 0.001).

We speculated that enhanced IFNβ expression in SF268 cells may relate to cell-type differences in IFNβ induction. To evaluate this hypothesis, RD and SF268 cells were seeded and infected with EV71 at MOIs of 1 and 40, and total protein was extracted to measure expression levels. Our results showed that viral protein expression levels were similar in RD cells infected with EV71 at an MOI of 1 and in SF268 cells infected at an MOI of 40 (Figure 5A). RT-qPCR was then performed to examine expression of vRNA and IFNβ. Although RD cells infected with EV71 at an MOI of 1 and SF268 cells infected with EV71 at an MOI of 40 expressed similar amounts of vRNA at 9 h p.i., 10 times more IFNβ transcripts were observed in EV71-infected SF268 cells (Figure 5A). These results indicate that SF268 cells are potent producers of IFN. Different amounts of EV71 vRNA were transfected into SF268 and RD cells, and we found that RD cells transfected with 0.25 μg EV71 vRNA yielded more virus RNA than did SF268 cells transfected with 0.5 μg EV71 vRNA (Figure 5B). However, much higher levels of IFNβ transcripts were detected in SF268 cells transfected with 0.5 μg EV71 vRNA than in RD cells transfected with 0.25 μg vRNA (Figure 5B). Therefore, in comparison to RD cells, SF268 cells may produce more IFNβ in response to virus insult.

To investigate which pathways are involved in efficient IFNβ gene induction in SF268 cells, TLR3, -8, and MDA-5 agonists were transfected into RD and SF268 cells for comparison. According to our previous data, the transfection efficiency for SF268 cells is approximately half that for RD cells, thus, different doses of agonists were utilized. SF268 and RD cells were treated with poly(A:U), a TLR3 agonist, for 12 h, and IFNβ expression levels were examined by RT-qPCR. Significantly more IFNβ transcripts were observed in SF268 cells than in RD cells (Figure 5C). Similarly, transfection of motolimod and poly(I:C)_HMW also induced a stronger IFNβ response in SF268 cells than in RD cells (Figure 5C). Next, the expression levels of TLR3, TLR8, and MDA-5 were assessed by RT-qPCR, revealing that mRNA expression of TLR3, TLR8, and MDA-5 was higher in SF268 cells than in RD cells (Figure 5D). In addition, the protein expression levels of TLR3, TLR8, and MDA-5 were examined by western blot analysis (Figure 5E). Our results revealed that higher amounts of TLR3 and MDA-5 proteins were expressed in SF268 cells than in RD cells.

### 3.6. EV71 Infection Induces Expression of ISGs

**Figure 6:**
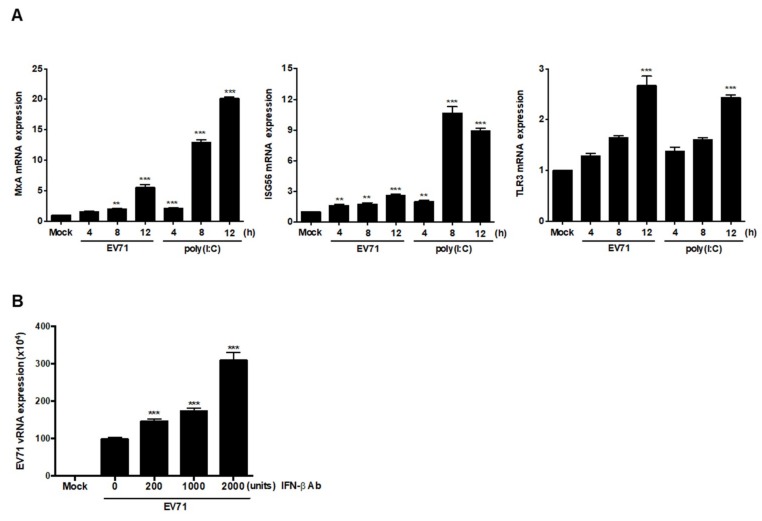
EV71 induces expression of interferon-stimulated genes (ISGs) in SF268 cells. (**A**) SF268 cells were infected with EV71 at an MOI of 40 or transfected with poly(I:C). Total RNA was extracted and the expression levels of MxA, ISG56, and TLR3 mRNA were determined by RT-qPCR. (**B**) SF268 cells were infected with EV71 at an MOI of 40 in the presence or absence of anti-IFNβ antibodies, after which expression of vRNA was analyzed by RT-qPCR. Asterisks indicate values that are statistically significant compared to mock-infected cells (*, *p* < 0.05, **, *p* < 0.01, ***, *p* < 0.001).

Secreted IFNβ can interact with IFNα/β receptors to activate expression of IFN-stimulated response element (ISRE)-containing genes. Neurons have been shown to be able to respond to IFNβ by upregulating transcription of CXCL10, CCL-5, and IRF7 [21], though a recent report showed that levels of IFNAR1 decrease in response to EV71 infection, which was attributed to inhibition of downstream ISG expression [8]. To examine whether expression of ISGs can be activated by EV71, SF268 cells were infected with EV71 or transfected with poly(I:C), and RT-PCR was applied to assess TLR3, ISG56, and MxA expression. Although the transcript levels of these three genes were increased in a time-dependent manner, we noticed that the increases in MxA and ISG56 levels in infected cells were less significant than those observed in cells stimulated with poly (I:C) (Figure 6A). To examine the roles of IFNβ in EV71-infected cells, secreted IFNβ was blocked by adding anti-IFNβ antibodies, and the expression of vRNA was analyzed by RT-PCR. Based on the results, IFNβ neutralization increased the expression levels of vRNA (Figure 6B). Therefore, IFNβ secreted by SF268 cells interacts with other cells to restrict viral growth. 

## 4. Discussion

Accumulating evidence demonstrates that neuronal cells can produce IFNβ in response to viral infections. For example, Theiler’s virus and La Crosse virus induce IFN production in the brain neurons of infected animals [20]. Moreover, differentiated NT2-N cells secrete IFNβ in response to rabies virus infection [21]. A recent study demonstrated that Sabin attenuated type 1 poliovirus-induced IFNβ expression in SK-N-SH cells [31]. However, knowledge regarding the abilities of other non-polio neurotropic enteroviruses to induce type I IFN production in human neuronal cells is limited. To the best of our knowledge, this is the first paper to show that EV71 can induce IFNβ expression in neural lineage cells.

Several members of the *Picornaviridae* family have evolved strategies to inhibit IFN production by interfering with the cascades involved in the induction of type I IFN expression [32,33], and thorough studies have been performed to investigate the inhibitory effect of EV71 on innate immune response regulation. It has recently been shown that EV71 3C^pro^ can cleave TRIF and thus suppress the transcription of IFNβ initiated by RIG-I recognition [25,26]. Furthermore, 2A^pro^ targets MAVS and thus reduces IFNβ expression [34]. A previous study also demonstrated that EV71 3C^pro^ inhibits the TLR3-mediated innate immune response by blocking TRIF [26]. The results of these studies suggest that EV71 viral proteins can efficiently suppress IFNβ induction in host cells. However, because we can observe enhanced expression of IFNβ transcripts in neural cells, we assumed that this phenomenon might be attributed to postponed expression of viral proteins in infected neural cells and efficient simulation of IFNβ transcription by vRNA. Nevertheless, other factors may also contribute to EV71-induced IFNβ upregulation in these specific host cells. 

The translation efficiency of enteroviruses is affected by both viral and cellular factors. It has been demonstrated that the lower translational efficiency of Sabin-PV correlates with its superior ability to induce type I IFN production in neuronal cells when compared to that of wild type PV [31]. Thus, IFNβ induction may not be obvious in RD cells because viral proteins are expressed in large amounts at the early stage of infection. In contrast, EV71 viral protein expression levels are significantly lower than those observed in RD cells. Accordingly, the low translation activity of viral proteins may be in part attributed to the significant IFNβ induction in EV71-infected SF268 cells.

TLR3 has been demonstrated to cause an antiviral response in bronchial epithelial cells infected with rhinovirus [35]. In addition, TLR3-mediated type I interferon signaling is important in limiting the replication of CVB3 in cells [36]. A recent study also demonstrated that silencing TLR3 impairs IFNβ expression in EV71-infected immune cells [37]. Except TLR3, EV71 infection has been shown to increase expression of TLR7 and -8 in brain tissues from fatal EV71 cases [38]. Additionally, TLR8 is associated with the cardiac inflammatory responses induced by infection with Coxsackie B viruses [39]. These findings indicate that TLR3 and TLR8 play roles in enterovirus infection. As our results reveal that TLR3 and TLR8 play essential roles in mediating IFN upregulation in SF268 cells, TLR3 and -8 may play essential roles in mediating EV71-induced IFNβ induction.

In addition to TLRs, neurons are equipped with functional RLRs such as RIG-I and MDA-5 [40]. Recent studies have shown that JEV infection activates neural production of proinflammatory cytokines including IL-6, IL-12p70, MCP-1, IP-10, and TNF-α via RIG-I-dependent pathways, and that ablation of RIG-I in neurons results in increased viral load [41]. Furthermore, Co et al. demonstrated that simian immunodeficiency virus (SIV) infection enhances expression of RIG-I and MDA-5 in the brains of infected monkeys [42]. Our knockdown experiments revealed that IFNβ mRNA expression is dependent on MDA-5, in accordance with the results obtained by Kuo et al. [43].

Differentiated neural cells are known to express more TLR3 than undifferentiated neural progenitors and thus evoke more potent immune responses [39]. Therefore, we hypothesized that enhanced IFNβ expression in neural cells may involve with IFNβ induction upon PAMP stimulation. Furthermore, our vRNA transfection experiments showed that when similar amounts of vRNA were detected in SF268 and RD cells, many more IFNβ transcripts were present in SF268 cells. Poly(A:U), motolimod, and poly(I:C)_HMW, agonists for TLR3, TLR8, and MDA-5, respectively, stimulate SF268 cells to produce more IFNβ transcripts in a dose-dependent manner. We also demonstrated that TLR3 and MDA-5 protein expression levels were significantly higher in SF268 cells than in RD cells. The discrepant PRR expression patterns between these two cell types may be associated with their different IFNβ induction capacities. Interestingly, our results showed that in addition to SF268 cells, other neural lineage cells are also capable of producing IFNβ when infected by EV71. Hence, these neural cells may also have superior ability to upregulate IFN expression. However, more experiments need to be performed to support this conclusion.

In summary, our results demonstrate that EV71 infection is able to upregulate expression of IFNβ in neural cells via TLR3, TLR8, and MDA-5, which may be associated with inefficient translation of EV71 RNA in neuronal cells and the superior ability of neural cells to produce IFNβ transcripts upon recognizing the virus. Additionally, IFNβ secreted by infected SF268 cells can interact with cells to limit viral growth, which is in accordance with previous observations [3,5,6,7]. Interestingly, MxA is the ISG that is able to regulate cell cycles [44]. A recent study showed the evidence that replication of EV71 could be affected by cell cycle arrest [45]. However, more experiments have to be performed to link cell duplication and anti-viral activities of IFNβ. The difference in IFNβ-inducing abilities in neural and RD cells reveals the unique properties of neural cells in restricting viral replication.

## Figures and Tables

**Table 1:** Primers used in this study.

Genes		Sequence (5′–3′)
EV71 5′UTR	Forward	CCC TGA ATG CGG CTA ATC C
	Reverse	ATT GTC ACC ATA AGC AGC CA
Human IFN	Forward	AGA AGG AGG ACG CCG CAT TG
	Reverse	TCA GTT TCG GAG GTA ACC TG
Human TLR3	Forward	AAG GGT GGC CCT TAA AAA TG
	Reverse	GTT TCC AGA GCC GTG CTA AG
Human TLR8	Forward	CAG AGC ATC AAC CAA AGC AA
	Reverse	GCT GCC GTA GCC TCA AAT AC
Human MDA5	Forward	AGG AGT CAA AGC CCA CCA TCT G
	Reverse	ATT GGT GAC GAG ACC ATA ACG GAT A
Human ISG56	Forward	TCT CAG AGG AGC CTG GCT AAG
	Reverse	CCA CAC TGT ATT TGG TGT CTA GG
Human MxA	Forward	TTC AGC ACC TGA TGG CCT ATC
	Reverse	TGG ATG ATC AAA GGG ATG TGG
Human 18S rRNA	Forward	GTA ACC CGT TGA ACC CCA TT
	Reverse	CCA TCC AAT CGG TAG TAG CG
